# Healthcare integration strategy implementation based on distance education and communication for health professionals in São Paulo City, Brazil: study protocol

**DOI:** 10.1186/1472-6963-14-S2-P9

**Published:** 2014-07-07

**Authors:** Mario Bracco, Alexandre Abdo, Marcelo Demarzo, Marcello Dalla, Fernando Colugnati, Ana Delgado, Ana Mafra

**Affiliations:** 1 Hospital Municipal Dr. Moyses Deutsch - M’Boi Mirim, São Paulo, SP, Brazil; 2 Hospital Israelita Albert Einstein, São Paulo, SP, Brazil; 3 State University of São Paulo, School of Medicine, São Paulo, SP, Brazil; 4 Federal University of São Paulo, Paulista School of Medicine, São Paulo, SP, Brazil; 5 Federal University of Juiz de Fora, School of Medicine, Juiz de Fora, MG, Brazil; 6 State University of Campinas, Geosciences Institute, Campinas, SP, Brazil

## 

The Brazilian healthcare system aims for universal access for the whole population, equity to prioritize health actions, and integrality in all assistance levels. The purpose of this research project is to promote and to evaluate intervention based on capacitation in clinical management focused on diseases that are part of the Brazilian list of ambulatory care sensitive conditions (ACSC). It will be delivered throughout a distance education course to health professionals who are based in a public hospitals and to 85 Family Health Strategy teams spread out over 18 Primary care Units (PCU), covering around 300,000 people, in the southern zone of São Paulo City. It will evaluate the use of communication tools, as a free internet-based platform and telemedicine, that will be made available to the health providers that can afford continuity and integrality of care to the patients who are followed by both health services. Also, health professionals learning and application of knowledge in the clinical practice as well as patient outcomes, will be evaluated.

Quasi-experimental cohort design with historical controls study of adult patients hospitalized by ACSC and will be followed up 1 year after the hospital discharge. Data collection will be performed on hospital and PCU patient’s health records and will be applied the Primary Care Attention Tool, the World Health Organization Quality of Life, and Sociodemographic questionnaires, to patients and health professionals, for social and environmental characterization, treatment plan adherence, disease monitoring and access to the health services. Data analysis will evaluate as outcomes the hospital readmissions of the followed patients, the use of the communication tools by the health providers, demographic, social and environmental variables, and hospitalization rates, patient’s time of hospitalization and mortality rates related to the patients.

This project is funded by the Brazilian Ministry of Health and São Paulo State Research Agency.

